# Novel Oxime Synthesized from a Natural Product of *Senecio nutans* SCh. Bip. (Asteraceae) Enhances Vascular Relaxation in Rats by an Endothelium-Independent Mechanism

**DOI:** 10.3390/molecules27103333

**Published:** 2022-05-22

**Authors:** Javier Palacios, Adrián Paredes, Marcelo A. Catalán, Chukwuemeka R. Nwokocha, Fredi Cifuentes

**Affiliations:** 1 Laboratorio de Bioquímica Aplicada, Química y Farmacia, Facultad de Ciencias de la Salud, Universidad Arturo Prat, Iquique 1110939, Chile; 2 Departamento de Química, Facultad de Ciencias Básicas, Universidad de Antofagasta, Antofagasta 1271155, Chile; 3 Laboratorio Química Biológica, Instituto Antofagasta (IA), Universidad de Antofagasta, Antofagasta 1271155, Chile; 4 Instituto de Fisiología, Facultad de Medicina, Universidad Austral de Chile, Valdivia 5090000, Chile; marcelo.catalan@uach.cl; 5 Department of Basic Medical Sciences, Faculty of Medical Sciences, The University of the West Indies, Mona Campus, Kingston 7, Jamaica; chukwuemeka.nwokocha@uwimona.edu.jm; 6 Laboratorio de Fisiología Experimental (EPhyL), Instituto Antofagasta (IA), Universidad de Antofagasta, Antofagasta 1271155, Chile; fredi.cifuentes@uantof.cl; 7 Departamento Biomédico, Facultad Ciencias de la Salud, Universidad de Antofagasta, Antofagasta 1271155, Chile

**Keywords:** natural products, oxime, vascular relaxation, endothelium, rat aorta

## Abstract

*Senecio nutans* Sch. Bip. and its constituents are reported to have antihypertensive effects. We isolated metabolite–1, a natural compound from *S. nutans* (4-hydroxy-3-(isopenten-2-yl)-acetophenone), and synthesized novel oxime – 1 (4-hydroxy-3-(isopenten-2-yl)-acetophenoxime) to evaluate their effect on vascular reactivity. Compounds were purified (metabolite–1) or synthetized (oxime–1) and characterized using IR and NMR spectroscopy and Heteronuclear Multiple Quantum Coherence (HMQC). Using pharmacological agents such as phenylephrine (PE) and KCl (enhancing contraction), acetylcholine (ACh), L-NAME (nitric oxide (NO) and endothelial function), Bay K8644-induced Ca_V1.2_ channel (calcium channel modulator), and isolated aortic rings in an organ bath setup, the possible mechanisms of vascular action were determined. Pre-incubation of aortic rings with 10^−5^ M oxime–1 significantly (*p* < 0.001) decreased the contractile response to 30 mM KCl. EC_50_ to KCl significantly (*p* < 0.01) increased in the presence of oxime–1 (37.72 ± 2.10 mM) compared to that obtained under control conditions (22.37 ± 1.40 mM). Oxime–1 significantly reduced (*p* < 0.001) the contractile response to different concentrations of PE (10^−7^ to 10^−5^ M) by a mechanism that decreases Cav1.2-mediated Ca^2+^ influx from the extracellular space and reduces Ca^2+^ release from intracellular stores. At a submaximal concentration (10^−5^ M), oxime–1 caused a significant relaxation in rat aorta even without vascular endothelium or after pre-incubate the tissue with L-NAME. Oxime–1 decreases the contractile response to PE by blunting the release of Ca^2+^ from intracellular stores and blocking of Ca^2+^ influx by channels. Metabolite–1 reduces the contractile response to KCl, apparently by reducing the plasma membrane depolarization and Ca^2+^ influx from the extracellular space. These acetophenone derivates from *S. nutans* (metabolite–1 and oxime–1) cause vasorelaxation through pathways involving an increase of the endothelial NO generation or a higher bioavailability, further highlighting that structural modification of naturally occurring metabolites can enhance their intended pharmacological functions.

## 1. Introduction

The species *Senecio nutans* Sch. Bip. (syn.: *Senecio graveolens* Wedd) (Asteraceae) is widely used as a medicinal plant by the Andean communities of northern Chile [1]. *S. nutans* is commonly called “chachacoma” and its infusion is used to treat gastric ulcers, [2], cold, bronchitis, whooping cough, asthma, and fever [3,4]. Moreover, chachacoma is used to treat problems associated with acute mountain sickness and to regulate arterial pressure [5]. Extracts and infusions of *S. nutans* have shown significant antioxidant, hemolytic, cytotoxic [2,6,7], vasodilatory, hypotensive, and antihypertensive activity in in vitro as well as animal models [8,9].

From a phytochemical point of view, analysis of the aerial parts of *S. nutans* show that p-hydroxyacetophenone and its benzophenone derivatives are the most abundant compounds [8,10,11]. Moreover, coumarins (such as scopoletin and scoparone) [6,7], the sesquiterpene lactone damsine, and the spirolactone canrenone and sesquiterpenes γ- and δ-cadinene [12] have been found in *S. nutans*.

One of the most studied metabolites of *S. nutans* is the p-hydroxyacetophenone derivative, 4-hydroxy-3-(3-methyl-2-butenyl) acetophenone, which displays significant antitumor activity in breast cancer cell lines [13], specific Gram-positive antimicrobial activity [14], and high antifungal activity against *Candida albicans* [14]. Additionally, this compound has antibacterial activity such as phytoalexins [15]. On the other hand, 4-hydroxy-3-(3-methyl-2-butenyl) acetophenone has demonstrated important dose-dependent vasodilator activity in rat aorta [8].

The vascular endothelium regulates the balance between vasodilator and vasoconstrictor tone under normal physiological conditions [16]. This mechanism involves the release of nitric oxide from endothelium, leading to the activation of vascular smooth muscle guanylyl cyclase (cGMP). cGMP induces the closure of voltage-dependent Ca^2+^ channels in plasma membranes and Ca^2+^ uptake by intracellular stores [17]. The function of the vascular endothelium has clinical implications in pathologies such as cardiovascular disease, diabetes, and obesity [18,19]. Regarding the antihypertensive mechanisms postulated for the drugs currently in use, more than half depend on the vascular endothelium, acting on the NO/cGMP pathway, as Ca^2+^ channel blockers or potassium channel activators [20].

Although somewhat forgotten, oximes have been reported to have a vasodilator effect in the presence and absence of vascular endothelium on isolated blood vessels [21]. In recent years, oximes have gained great interest because of the ease of synthesis from carbonyl compounds, aldehydes, and ketones [22], and their enormous potential biological activity [23], especially in their roles such as being donors of nitric oxide [24], as well as potential β1 and β2 adrenergic receptor antagonists, which might be useful for the treatment of arrhythmia and pulmonary arterial hypertension [25]. In the search for new derivatives of natural products with potential cardiovascular properties, we have prepared a new oxime (not reported in the scientific literature) from the natural compound 4-hydroxy-3-(3-methyl-2-butenyl) acetophenone. In this study, we have evaluated the vascular response in a rat aortic model to understand the mechanisms of vascular reactivity of both compounds—a natural product and an oxime.

## 2. Results

### 2.1. Isolation of Natural Products from S. nutans and Oxime Synthesis

**4-hydroxy-3-**(**isopenten-2-yl**)**-acetophenone** (Metabolite–1). This natural compound was isolated from *S. nutans*, identified in a previous study [8]. Crystalline solid; mp: 90–92 °C; HRMS *m/z* 204.150 (C_13_H_16_O_2_, [M^+^]); IR (KBr) 3000–3500, 1750–2000, 1645, 1350–1550 cm^−1^. 

**4-hydroxy-3-**(**isopenten-2-yl**)**-acetophenoxime** (Oxime–1) crystallizes from dichloromethane as green crystals, m.p. 118–120 °C molecular weight 219, which is assignable to C_13_H_17_NO_2._ The IR spectrum shows bands of low intensity between 1750–2000 cm^−1^ and 1350–1550 cm^−1^, assignable to the presence of a benzene ring; a wide band between 3000–3500 cm^−1^, associated with the presence of vibrations of a –C=N–OH group and hydroxyl group; and an intense band at 1602 cm^−1^, resulting from –C=N–OH interactions of oximes. These last two sets of signals confirm the modification of the carbonyl group in the reaction. The ^1^H and ^13^C NMR spectra show signals assignable to 15 hydrogen atoms and 13 carbon atoms, respectively. The chemical shifts and the DEPT spectrum indicate the presence of three CH_3_, one CH_2_ δ to the aromatic ring, three aromatic CHs, one olefinic CH, and 5 quaternary carbons of the sp^2^ type. A methyl group is unique to the ketoxime group (CH_3_–C=N). This methyl is displaced towards the higher field at δ 2.26 ppm, with respect to the precursor metabolite–1 δ 2.60 ppm. The remaining two methyls are vinyl (Table 1 and Table 2).

Metabolite–1 and oxime–1 were identified by 1D and 2D NMR, DEPT, HMBC, HMQC, FT-IR, and UV spectra (Supplementary Materials, Figures S1–S13).

A signal can be observed in the ^13^C NMR spectrum that resonates at δ 155.91 ppm and corresponds to a carbon with double bond to nitrogen (C=N) characteristic of oximes; this new signal replaces the signal that resonated at δ 198 ppm (C-12) and that corresponded to the carbonyl group of metabolite–1. All the above information confirms the formation of the reaction product. 

**Figure 1:**
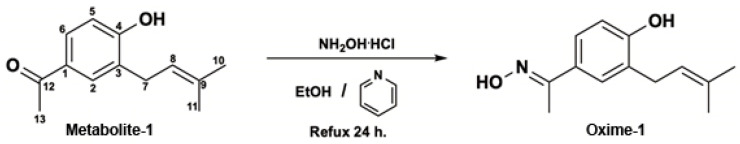
Structure and synthetic scheme for the preparation of new oxime 4-hydroxy-3-(isopenten-2-yl)-acetophenoxime (Oxime–1) from secondary metabolite 4-hydroxy-3-(isopenten-2-yl)-acetophenone (Metabolite–1).

**Figure 2:**
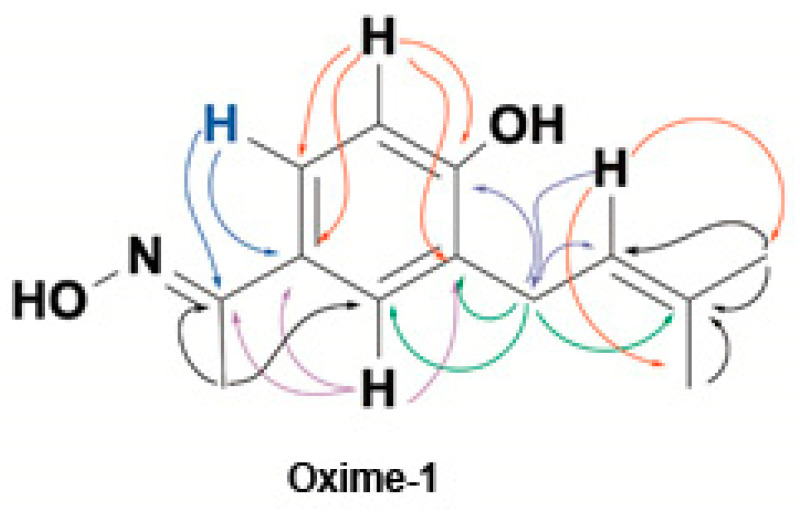
Selected HMBC correlations for 4-hydroxy-3-(isopenten-2-yl)-acetophenoxime (Oxime–1).

In the Heteronuclear Multiple Quantum Coherence (HMQC) spectrum, the same couplings are observed as for the precursor (metabolite–1), which is reasonable as the structural modification has been made on the carbonyl group and does not affect the rest of the compound’s structure. The analysis of the spectroscopic information has allowed us to establish that the compound obtained after the reaction corresponds to 4-hydroxy-3-(isopenten-2-yl)-acetophenoxime (Oxime–1; Figure 1). The coupling patterns deduced from the HMBC spectrum are indicated in Figure 2.

### 2.2. Effect of Metabolite–1 and Oxime–1 on Vascular Relaxation: Role of the Endothelium

**Figure 3:**
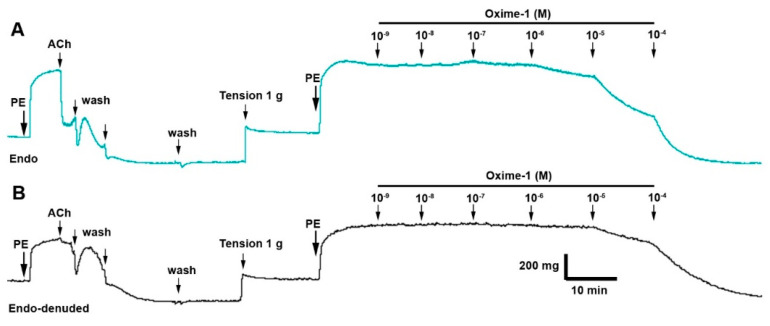
Oxime–1 causes relaxation in rat aorta via the endothelium-dependent pathway. Aortic rings were pre-contracted with 10^−6^ M phenylephrine (PE), and cumulative concentrations of oxime–1 (10^−9^ to 10^−4^ M) were added in bath. Representative trace showing of vascular effect in intact aortic ring (Endo) (**A**) and endothelium-denuded (Endo-denuded) aortic ring (**B**).

**Figure 4:**
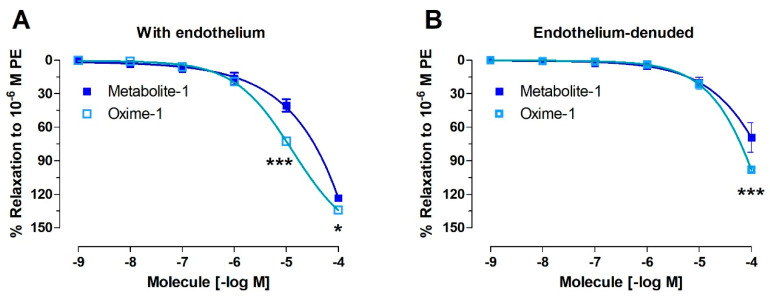
Metabolite–1 and oxime–1 cause relaxation in rat aorta via the endothelium-dependent pathway. Aortic rings were pre-contracted with 10^−6^ M PE, and cumulative concentrations of molecules (10^−9^ to 10^−4^ M) were added in bath. The protocol was repeated in intact rat aorta (**A**), and endothelium-denuded aorta (**B**). Data are presented as the average standard error of the mean (SEM) of 4–6 independent experiments. * *p* < 0.05; *** *p* < 0.001.

Both compounds caused vascular relaxation in PE-treated intact rat aorta: 41 ± 6% metabolite–1, 73 ± 2% oxime–1 (10^−5^ M; Figure 3A and Figure 4A). Interestingly, oxime–1 caused a higher relaxation than metabolite–1 (*p* < 0.001). As shown in Table 3, the half-maximal effective concentration (EC_50_) to oxime–1 (10.14 ± 8.46 μM) was significantly lower (*p* < 0.001) compared to metabolite–1 (24.44 ± 7.98 μM). 

The denudation of endothelium in aortic rings provoked a drastic decreased of relaxation, 30% at 10^−5^ M concentration for both molecules. Thus, the relaxation was approximately 20% in endothelium-denuded rat aorta: 22 ± 4% oxime–1 versus 20 ± 5% of metabolite–1, (10^−5^ M; Figure 3B and Figure 4B). 

**Figure 5:**
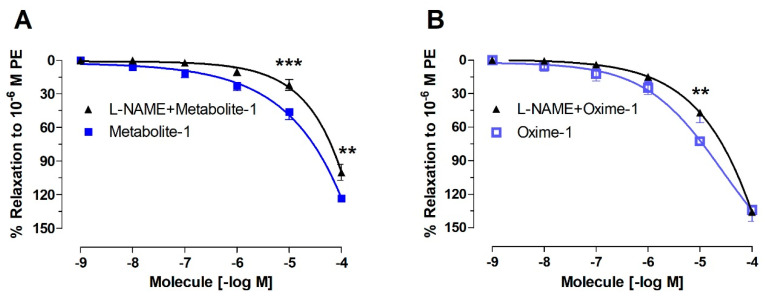
Metabolite- and oxime- caused relaxation in rat aorta depends on the nitric oxide pathway (**A**,**B**). Intact aortic rings were pre-incubated with 10^−4^ M L-NAME for 20 min. Subsequently, aortic rings were pre-contracted with 10^−6^ M PE, and cumulative concentrations of molecules (from 10^−9^ to 10^−4^ M) were added in bath. Data are presented as the average standard error of the mean (SEM) of 4–5 independent experiments. ** *p* < 0.01; *** *p* < 0.001.

To evaluate whether the release of endothelial factors was involved, the vascular tissue was pre-incubated with an inhibitor of endothelial nitric oxide synthase (NOS) [26]. We found that the pre-incubation of the intact aortic rings with 10^−4^ M L-NAME significantly decreased relaxation to 10^−5^ M concentration: metabolite–1, 41 ± 6% vs. 22 ± 5% L-NAME, *p* < 0.001 (Figure 5A); oxime–1, 73 ± 2% vs. 42 ± 9% L-NAME, *p* < 0.01 (Figure 5B).

Our observations were confirmed by the significant differences in the EC_50_ in the presence and absence of L-NAME for all molecules (Table 3). 

On the other hand, the pre-incubation with L-NAME partially blunted the relaxation of metabolite–1 in endothelium-denuded aortas, whereas oxime–1 did not. As shown in Table 3, the EC_50_ to oxime–1 was significantly lower (*p* < 0.01) in the presence of L-NAME (26.49 ± 7.97 μM) compared to endothelium-denuded aortic rings (81.88 ± 7.79 μM), although it was significantly higher than the EC_50_ obtained in intact aortas treated with oxime–1.

### 2.3. Effect of Metabolite–1 and Oxime–1 on the Contractile Response to KCl: Role of External Ca^2+^

**Figure 6:**
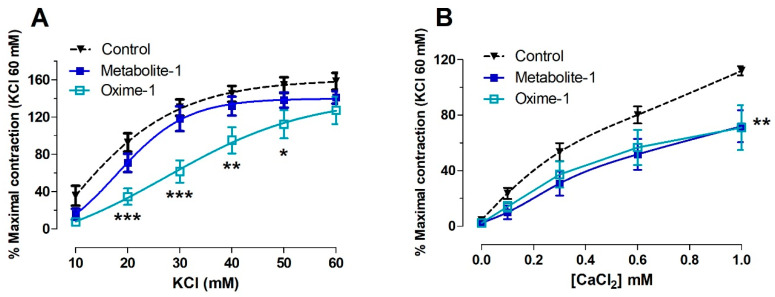
Effect of metabolite–1 and oxime–1 on the contractile response to KCl in rat aorta. The contraction to KCl was compared to contraction with KCl 60 mM obtained at the beginning of the experiment, during the equilibration period of tissues. Intact aortic rings were pre-incubated with 10^−5^ M metabolite–1 or oxime–1 for 20 min. Subsequently, aortic rings were contracted with cumulative concentrations of KCl (10 to 60 mM) (**A**). On the other hand, the aortic rings were pre-incubated in a free Ca^2+^ buffer for 10 min before 40 mM KCl was added, and then, the CaCl_2_ (0.1 to 1 mM) was added to the bath (**B**). Data are presented as the average standard error of the mean (SEM) of 4 independent experiments. * *p* < 0.05; ** *p* < 0.01; *** *p* < 0.001 versus the curve in the absence of bioactive molecules (Control).

To analyze whether the relaxation effect of molecules is mediated by the membrane depolarization, the contractile response to KCl was tested. In contrast to the vascular relaxation by both compounds mentioned above, oxime–1 significantly reduced the contractile response to KCl, whereas metabolite–1 did not it. The results showed that the pre-incubation of aortic rings with 10^−5^ M oxime–1 significantly (*p* < 0.001) decreased the contractile response to 30 mM KCl compared to the intact aorta: 130 ± 9% control versus 62 ± 9% oxime–1 (Figure 6A). The EC_50_ to KCl significantly (*p* < 0.01) increased in the presence of oxime–1 (37.72 ± 2.10 mM) versus control (22.37 ± 1.40 mM; Table 4). 

Afterwards, we determined the role of extracellular Ca^2+^ in the contractile response to KCl. First, the aortic rings were pre-incubated with metabolite–1 or oxime–1 (10^−5^ M) in Ca^2+^-free medium. Second, the vascular tissue was stimulated with KCl (40 mM) to induce a contractile response while adding a cumulative concentration of extracellular Ca^2+^ (0.1 to 1.0 mM). In a Ca^2+^-free medium, the results confirmed that metabolite–1 (72 ± 11%), and oxime–1 (71 ± 16%) significantly (*p* < 0.01) decreased the contractile response to a submaximal KCl concentration (40 mM) compared to intact aorta (112 ± 3% control; 1.0 mM CaCl_2_; Figure 6B). 

### 2.4. Effect of Metabolite–1 and Oxime–1 on the Contractile Response to PE and Extracellular Ca^2+^ Influx

The next step was to determine if the relaxation induced by metabolite–1 and oxime–1 can be mimicked in a receptor-mediated contractile response. In this line, the contractile response to PE was tested and both compounds significantly reduced the contractile response to PE, although oxime–1 showed a more highly significant (*p* < 0.001) reduction of the contractile response to different concentrations of PE (10^−7^ to 10^−5^ M).

**Figure 7:**
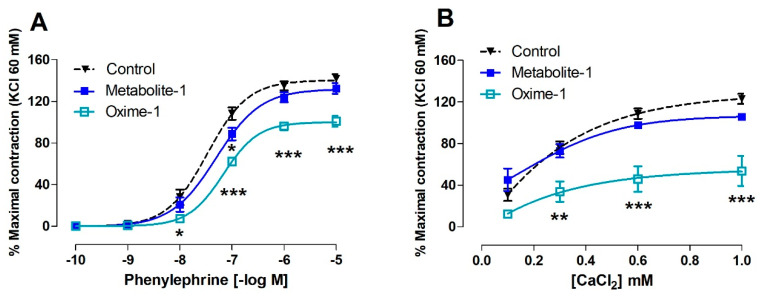
Effect of metabolite–1 and oxime–1 on the contractile response to PE in rat aorta. The contraction to PE was compared to contraction with KCl 60 mM obtained at the beginning of the experiment, during the equilibration period of tissues. Intact aortic rings were pre-incubated with 10^−5^ M metabolite or oxime for 20 min. Subsequently, aortic rings were contracted with cumulative concentrations of PE (10^−10^ to 10^−5^ M) (**A**). In addition, the aortic rings were pre-incubated in a Ca^2+^-free buffer for 10 min before 10^−7^ M PE was added, and then, the CaCl_2_ (0.1 to 1 mM) was added to the bath (**B**). Data are presented as the average standard error of the mean (SEM) of 4 independent experiments. * *p* < 0.05; ** *p* < 0.01; *** *p* < 0.001 versus the curve in the absence of bioactive molecules (control).

The results showed that the pre-incubation of aortic rings with 10^−5^ M oxime–1 significantly (*p* < 0.001) decreased the contractile response to 10^−7^ M PE (62 ± 3% oxime–1) compared to the intact aorta (108 ± 6% control), whereas metabolite–1 did not it (Figure 7A). Surprisingly, the attenuated contraction in presence of oxime–1 was maintained even with higher concentrations of PE (10^−7^ to 10^−5^ M). Significantly increased EC_50_ to PE confirmed the adrenergic vascular response observed in the presence of oxime–1 (67.28 ± 8.67 nM; *p* < 0.05) versus control (34.04 ± 8.49 nM; Table 5). 

In a Ca^2+^-free medium, the results confirmed that oxime–1 (54 ± 14%) significantly (*p* < 0.001) decreased the contractile response to PE (10^−7^ M) compared to intact aorta (123 ± 5% control; 1.0 mM CaCl_2_; Figure 7B). No changes were found in EC_50_ for either bioactive molecule. 

### 2.5. Effect of Metabolite–1 and Oxime–1 on the Contractile Response to KCl and Bay K8644

**Figure 8:**
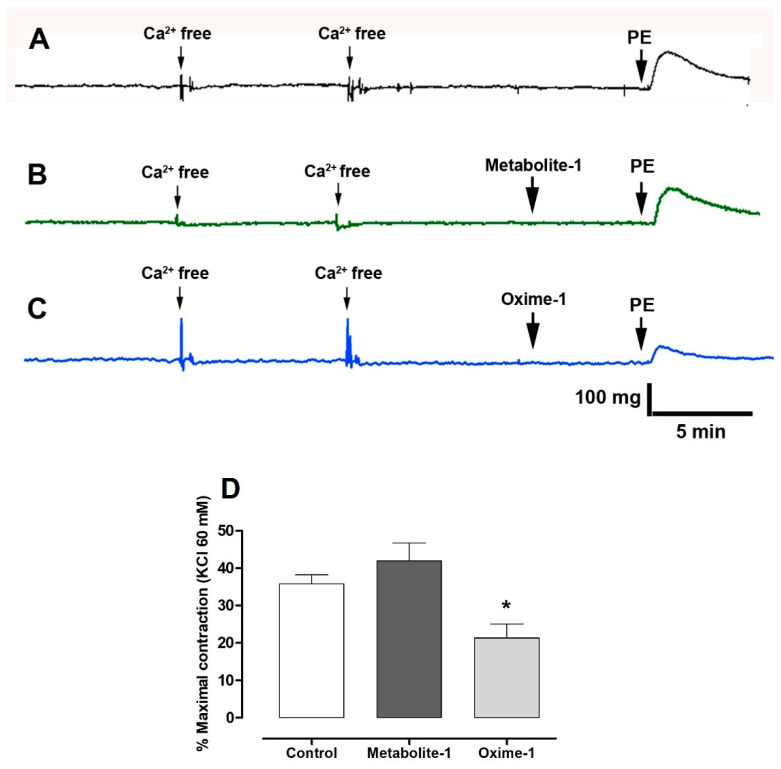
Effect of metabolite–1 and oxime–1 on the contractile response to PE in Ca^2+^-free medium (**A**). Intact aortic rings were used as a control (**A**), or pre-incubated with 10^−5^ M of metabolite–1 (**B**) or oxime–1 (**C**) for 20 min, and then 10^−7^ M PE was added in the Ca^2+^-free medium. Data are presented as the average standard error of the mean (SEM) of 4 independent experiments. * *p* < 0.05 versus control (**D**).

Figure 8 shows that the pre-incubation with oxime–1 (21 ± 4%; *p* < 0.05) significantly decreased the adrenergic contraction induced by releasing of intracellular Ca^2+^ in Ca^2+^-free medium compared to intact aorta without oxime–1 (36 ± 2% control). However, metabolite–1 did not decrease the contractile response by releasing intracellular Ca^2+^.

**Figure 9:**
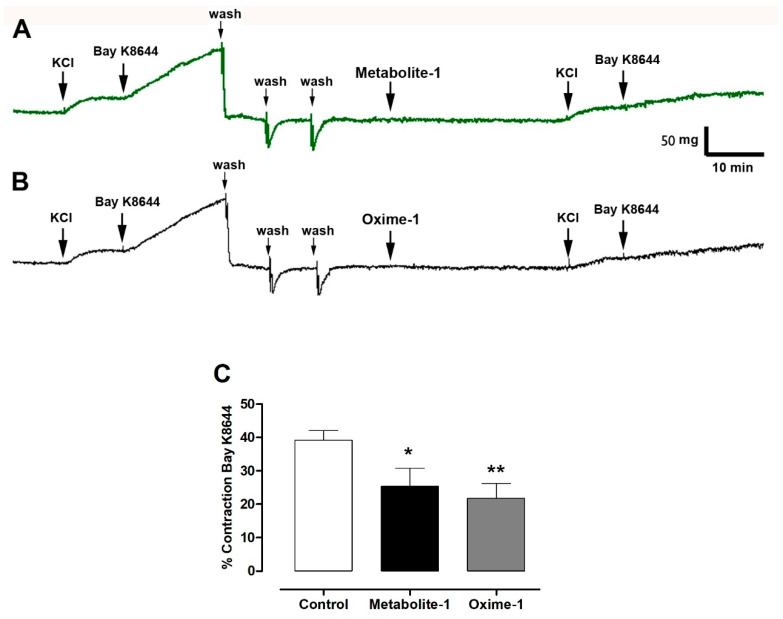
Metabolite–1 and oxime–1 decreases contractile response to Bay K8644 in normal Ca^2+^ buffer. Intact aortic rings were contracted with 10 mM KCl, and 10 nM Bay K8644 in the presence of 10^−5^ M metabolite–1 (**A**) or 10^−5^ M oxime–1 (**B**) for 20 min. Data are presented as the average standard error of the mean (SEM) of 4 independent experiments (**C**). * *p* < 0.05, ** *p* <0.01 versus control (without bioactive molecules).

Conversely, both compounds significantly reduced the contraction induced by Bay K8644, which promotes Ca^2+^ influx across the plasma membrane by opening Ca_V1.2_ channels. The effect of oxime–1 was higher compared to that displayed by metabolite–1: 39 ± 3% control vs. 25 ± 5% metabolite–1 (*p* < 0.05) and 22 ± 4% oxime–1 (*p* < 0.001; Figure 9). 

## 3. Discussion

In the present study, we show for the first time that synthesized oxime from metabolite–1, a natural product isolated from *Senecio nutans* Sch. Bip., exerts a substantial relaxation effect in rat aorta. Metabolite–1 and oxime–1 cause a dose-dependent relaxation of the vascular endothelium, involving endothelial nitric oxide release. Although both compounds reduce the contractile response by decreasing Ca^2+^ influx from the extracellular space induced by Cav_1.2_ channel opening, the effect of oxime–1 was also due to reduction of Ca^2+^ released from intracellular stores. 

Oxime–1 was more effective than metabolite–1 in relaxing pre-contracted intact rat aorta with submaximal concentrations of KCl or PE. Together, the chemical modification of metabolite–1 to oxime–1 significantly enhanced the relaxation effect on the vasculature. 

Denudation of the endothelium in aortic rings reduced the relaxation induced by both compounds in response to submaximal concentrations of KCl and PE. These data suggest that both compounds produce relaxation in intact rat aorta, in part, mediated by the release of factors from the vascular endothelium. Indeed, pre-incubation of the vascular tissue with inhibitor of endothelial nitric oxide synthase (L-NAME) reduced the relaxation induced by metabolite–1 and oxime–1, strongly suggesting that the nitric oxide (NO) pathway is involved in the vascular response promoted by metabolite–1 and oxime–1, in agreement with previous studies showing the relaxation effects of oximes on isolated blood vessels with denuded endothelium and endothelium [21]. Oximes possess a R_2_C=NOH group, which is metabolized by hemoproteins, hemoglobin, and catalase, generating NO and vasodilatation [24,27].

Interestingly, at a maximal concentration, oxime–1 caused a significant relaxation in rat aorta even without vascular endothelium or after pre-incubating the tissue with L-NAME. Vascular reactivity of the tissue was recovered after washing. Other studies demonstrated that L-NAME did not reduce the relaxation effect of oxime derivatives, but inhibitors of soluble guanylyl-cyclase did [21]. This finding suggests that oxime–1 could be a potential bioactive molecule in an injured vascular endothelium [23]. 

Metabolite–1 and oxime–1 present 4-hydroxyacetophenone moiety in their backbone (see Figure 1). Previous studies showed that acetophenone derivatives, such as 4-hydroxyacetophenone, from *Cynanchum wilfordii* (Maxim.) Hemsl. ameliorates the vascular endothelial dysfunction by improvement of the NO/cGMP pathway in rat aorta [28,29]. Another study reported vasodilation mechanism associated with acetophenone derivatives (i.e., apocynin) that inhibit NADPH oxidase activity, leading to the generation of lower amounts of reactive oxygen species (ROS) [30]. Therefore, these findings could provide support for the vasodilator effect of these acetophenone derivates (metabolite–1 and oxime–1), involving an increase of the endothelial NO generation or a higher bioavailability due to NADPH oxidase inhibition [8,31].

In the contraction studies approach, the vascular contractile response was studied by membrane depolarization with KCl and by the activation of G-protein coupled receptor with PE. We found that oxime–1 counteracted the contraction in aortic rings induced by low and intermediate concentrations of KCl, but not at the maximal concentration of KCl. Conversely, although metabolite–1 did not decrease the contractile response to KCl in normal medium, both compounds under Ca^2+^-free conditions decreased the contraction induced by KCl or voltage-dependent Ca^2+^ channel type-L opening induced by Bay K8644. 

In a same line of the drug potency, the contractile response to PE significantly decreased with pre-incubation of oxime–1 in normal and Ca^2+^-free medium, but not with metabolite–1. Reduction of the contractile response to KCl in the presence of oxime–1 can be considered moderate compared to PE. In fact, the attenuated contraction in the presence of oxime–1 was maintained even with maximal concentration of PE, but not with KCl. 

Our data suggests that oxime–1 reduces the vascular contraction because it decreases Ca^2+^ release from intracellular stores as well as Ca^2+^ influx from extracellular space. This is important because the Ca^2+^ release from intracellular stores is the first step in triggering Ca^2+^ influx through Ca^2+^ channels of the plasma membrane and produces arterial vasocontraction [32]. First, we observed a low contractile response to PE in the presence of oxime–1 in Ca^2+^-free medium, before adding Ca^2+^ to the bath. Second, there was a small increase in contractile response after adding Ca^2+^.

Metabolite–1 and oxime–1 present isopentenyl residue in their backbone. It is possible that isopentenyl derivatives affect the vascular response. In fact, isopentenyl derivative causes a selective inhibition of Ca^2+^ influx through single Transient Receptor Potential Cation Channel (TRPV3) in endothelial cells of cerebral parenchymal arterioles and uterine radial arteries in rats [33,34]. On the other hand, an activated TRPV1 channel causes vascular smooth muscle contraction due to Ca^2+^ influx via TRPV1, and the contraction is amplified by depolarization-induced Ca^2+^ influx via Cav_1.2_ in the plasma membrane of the coronary arteries [35]. 

Previous studies reported that nifedipine, an antagonist of voltage-dependent Ca^2+^ channels, blunted the relaxation caused by 4-hydroxyacetophenone in rat aorta pre-contracted with PE, suggesting that voltage-dependent Ca^2+^ channels are involving in the relaxation effect of acetophenone derivates, such as metabolite–1 and oxime–1 [36].

## 4. Materials and Methods

### 4.1. Chemicals

Hydroxylamine hydrochloride, pyridine, magnesium sulphate, L-phenylephrine hydrochloride (PE), acetylcholine chloride (ACh), N^G^-nitro-l-arginine methyl ester (L-NAME), and (±)-Bay K8644 were bought from Sigma-Aldrich (St. Luis, MO, USA). The metabolite and oxime were dissolved in DMSO (0.1% final concentration). 

### 4.2. Isolation of Natural Products from S. nutans

The natural product 4-hydroxy-3-(isopenten-2-yl)-acetophenone (Metabolite–1) was isolated from *S. nutans* according to a previous protocol [8]. Briefly, the hydroalcoholic extract was re-suspended in distilled water and extracted successively with n-hexane and chloroform. Metabolite–1 was isolated from chloroform sub-fraction. The organic solutions were concentrated on a rotary evaporator and lyophilized. The structural elucidation was carried out using the spectroscopic data. 

### 4.3. Synthesis of Oxime

Synthesis of oxime was performed as previously described with a few modifications [37]. To a solution of the keto-ester (500 mg, 2.5 mmol, 1.0) and hydroxylamine (180 mg, 2.5 mmol, 1.0), ethanol (10 mL) was added pyridine (1.6 mL × mmol) in 1 portion. The reaction mixture was heated at 65°C for 24 h and then concentrated on a rotary evaporator. The residue was partitioned between ether (50 mL) and water (10 mL). The organic layer was sequentially washed with HCl (0.5 N, 10–50 mM) and water (10 mL), and then dried over MgSO_4_. Concentration in vacuo provided the oxime as a 3:1 mixture of E:Z isomers as a colorless solid.

### 4.4. Animals

Male Sprague Dawley rats (6–8 weeks old; *n* = 12) weighing between 170 g and 200 g were used in this study. The investigation was conducted in accordance with the local animal research committee of Universidad de Antofagasta (CEIC #275/2020). The animals were housed in plastic cages at room temperature (22–25 °C) and a humidity of 45–51% and had full access to tap water and food (ad libitum). They were randomized and assigned into the different groups tested.

### 4.5. Isolation of Rat Aorta and Vascular Reactivity Assays

This procedure was performed based on the method previously described [34]. Animals were euthanized by cervical dislocation. The aortic rings were placed in an organ bath with Krebs–Ringer bicarbonate (KRB) solution (in mM): 4.2 KCl, 1.19 KH_2_PO_4_, 120 NaCl, 25 NaHCO_3_, 1.2 MgSO_4_, 1.3 CaCl_2_, and 5 D-glucose, with pH 7.4, 37 °C, 95% O_2_, and 5% CO_2_. After the equilibration period for 30 min, the aortic rings were stabilized by three successive near-maximum contractions with KCl (60 mM) for 10 min. The passive tension on aorta was 1.0 g, which was determined to be the resting tension for obtaining maximum active tension induced by 60 mM KCl [38].

To evaluate the contractile response to phenylephrine (PE, 10^−10^ to 10^−5^ M) or KCl (10 to 60 mM), the tissue was pre-incubated in KRB for 20 min prior to contraction. In another applied protocol, the relaxation capacity of the extract or isolated metabolite was determined. In this case, the tissue was pre-contracted with 10^−6^ M PE, and increasing concentrations of bioactive molecules were added to the organ bath on the vascular plateau response. The integrity of the vascular endothelium was evaluated with 10^−6^ M acetylcholine (ACh) at the beginning of the experiment. 

### 4.6. Statistical Analysis

The results obtained from the experiments are expressed as mean ± standard error of the mean. Statistical analysis of the data was performed using analysis of variance (two-way ANOVA) followed by Bonferroni post hoc test. In addition, the determination of the sensitivity (EC_50_ or IC_50_) was performed using nonlinear regression (sigmoidal) via Graph Pad Prism software, version 5.0. (GraphPad Software, Inc., La Jolla, CA, USA). Statistical significance was set at *p* < 0.05.

## 5. Conclusions

**Figure 10:**
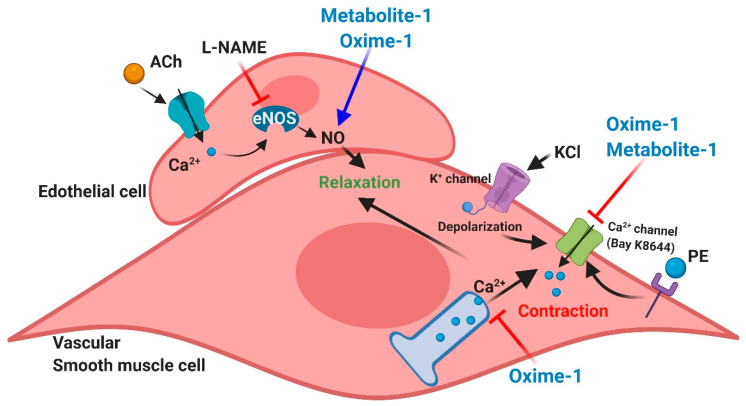
Putative model of vascular relaxation in rat blood vessel induced by oxime–1 and metabolite–1. Phenylephrine (PE) selectively stimulates the alpha-adrenergic receptor in vascular smooth cells, leading to vascular contraction by releasing Ca^2+^ from intracellular stores and increased Ca^2+^ influx from the extracellular space. KCl-induced contraction is due to the plasma membrane depolarization and Ca^2+^ influx. Oxime–1 decreases the contractile response to PE by blunting the release of Ca^2+^ from intracellular stores and blocking Ca^2+^ influx by channels. Metabolite–1 reduces the contractile response to KCl, in part, because of the reduction of depolarization of plasma membrane and Ca^2+^ influx from the extracellular space. Both bioactive molecules cause relaxation in rat aorta in an endothelium-dependent manner, through endothelial nitric oxide (NO).

As shown in Figure 10, these acetophenone derivates (metabolite–1 and oxime–1) cause vasorelaxation through pathways involving an increase in endothelial NO levels or a higher bioavailability. Oxime–1 caused significant relaxation of isolated vessels even without vascular endothelium or after inhibiting the nitric oxide generation. Our data shows a substantial effect of oxime–1 on the contractile response induced by PE compared to metabolite–1. Oxime–1 decreased the contractile response to PE by blunting the release of Ca^2+^ from intracellular stores and apparently blocking Ca^2+^ influx by channels [39]. Metabolite–1 reduced the contractile response to KCl, in part, because of the reduction of depolarization of plasma membrane and Ca^2+^ influx from the extracellular space [40]. Both bioactive molecules caused relaxation in rat aorta in an endothelium-dependent manner, through endothelial nitric oxide (NO). Results from this approach to improve the vasodilator effect of bioactive molecules from *S. nutans* through chemical modification of natural products will provide new molecules for treating hypertension.

## Figures and Tables

**Table 1:** ^1^H NMR spectroscopic data of metabolite–1 and respective oxime–1 (in CDCl_3_ at 400 MHz).

Position	Metabolite–1	Oxime–1
	*δ*H (ppm) *J* in Hz	*δ*H (ppm) *J* in Hz
1	----	----
2	7.79 (2.2)	7.40
3	----	----
4	----	----
5	6.91 (d), (8.3)	6.79 (d), (8.3)
6	7.80 (dd), (8.4–2.4)	7.37
7	3.44 (d), (8.2)	3.37 (d), (7.1)
8	5.36	5.31 (t), (6.2)
9	----	----
10	1.82 (d), (1.2)	1.78 (s)
11	1.82 (d), (1.2)	----
12	----	----
13	2.60 (s)	2.26 (s)
OH	6.57 (s)	----

**Table 2:** ^13^C NMR spectroscopic data of metabolite–1 and respective oxime–1 (in CDCl_3_ at 150 MHz).

Position	Metabolite–1	Oxime–1
	*δ*H (ppm)	*δ*H (ppm)
C1	159.71	115.47
C2	130.91	127.790
C3	127.79	126.93
C4	129.72	129.14
C5	115.40	115.72
C6	128.95	125.52
C7	29.11	29.87
C8	121.33	121.45
C9	134.64	135.15
C10	25.80	25.77
C11	17.91	17.90
C12	198.56	155.91
C13	26.29	12.12

**Table 3:** Relaxation effect of metabolite–1 or oxime–1 in pre-contracted aortic rings with PE: in presence, absence of endothelium, and pre-incubated with 10^−4^ M L-NAME.

Molecule	Endothelium	Endothelium-Denuded	L-NAME
Metabolite–1	24.44 ± 7.98	53.34 ± 4.56	66.32 ± 7.22 *
Oxime–1	10.14 ± 8.46 ^###^	81.88 ± 7.79 ***	26.49 ± 7.97 **^,$$^

The values represent the half maximal effective concentration (EC_50_ μM) to different bioactive molecules. The values are mean ± standard error of the mean (SEM) of 4–6 independent experiments. Statistically significant difference: * *p* < 0.05; ** *p* < 0.01; *** *p* < 0.001 vs. Endothelium; ^###^ *p* < 0.001 vs. Metabolite–1; ^$$^ *p*< 0.01 vs. endothelium-denuded aorta.

**Table 4:** Effect of metabolite–1 or oxime–1 on contractile response to KCl (10 to 60 mM) in intact aortic rings incubated under different Ca^2+^ concentrations in the external medium.

Molecule	KCl (mM)	CaCl_2_ (mM)
Control	22.37 ± 1.40	0.39 ± 0.03
Metabolite–1	21.98 ± 1.20	0.41 ± 0.06
Oxime–1	37.72 ± 2.10 ***	0.33 ± 0.07

The values represent the half maximal effective concentration (EC_50_ μM) to KCl or CaCl_2_. The values are mean ± standard error of the mean (SEM) of 4 independent experiments. Statistically significant difference: *** *p* < 0.001 control vs. oxime–1.

**Table 5:** Effect of metabolite–1 or oxime–1 on contractile response to PE (from 10^−10^ to 10^−5^ M) in intact aortic rings incubated in normal medium or Ca^2+^-free medium.

Molecule	PE (nM)	CaCl_2_ (mM)
Control	34.04 ± 8.49	0.32 ± 0.03
Metabolite–1	49.77 ± 8.44	0.33 ± 0.04
Oxime–1	67.28 ± 8.67 *	0.33 ± 0.10

The values represent the half maximal effective concentration (EC_50_ μM) to PE or CaCl_2_. The values are mean ± standard error of the mean (SEM) of 4 independent experiments. Statistically significant difference: * *p*< 0.05 vs. control.

## Data Availability

Not applicable.

## Supplementary Materials

The following supporting information can be downloaded at: https://www.mdpi.com/article/10.3390/molecules27103333/s1, The spectroscopy information of both compounds Figure S1–S13.
