# Supplementary material for: Novel Oxime Synthesized from a Natural Product of Senecio nutans SCh. Bip. (Asteraceae) Enhances Vascular Relaxation in Rats by an Endothelium-Independent Mechanism
Source: Molecules. 2022 May 22;27(10):3333. doi: 10.3390/molecules27103333 (PMC9146311; doi:10.3390/molecules27103333)
Supplement: Supplementary file 1 [file molecules-27-03333-s001.zip › molecules-1732287-supplementary.pdf]

Supplementary materials

Novel Oxime Synthesized from a Natural Product of *Senecio nutans* SCh. Bip. (Asteraceae) Enhances Vascular Relaxation in Rats by an Endothelium-Independent Mechanism

Javier Palacios [1,*], Adrián Paredes [2,3,*], Marcelo A. Catalán [4], Chukwuemeka R. Nwokocha [5] and Fredi Cifuentes [6,7]

[1] Laboratorio de Bioquímica Aplicada, Química y Farmacia, Facultad de Ciencias de la Salud, Universidad Arturo Prat, Iquique 1110939, Chile
[2] Departamento de Química, Facultad de Ciencias Básicas, Universidad de Antofagasta, Antofagasta 1271155, Chile
[3] Laboratorio Química Biológica, Instituto Antofagasta (IA), Universidad de Antofagasta, Antofagasta 1271155, Chile
[4] Instituto de Fisiología, Facultad de Medicina, Universidad Austral de Chile, Valdivia 5090000, Chile; marcelo.catalan@uach.cl
[5] Department of Basic Medical Sciences, Faculty of Medical Sciences, The University of the West Indies, Mona Campus, Kingston 7, Jamaica; chukwuemeka.nwokocha@uwimona.edu.jm
[6] Laboratorio de Fisiología Experimental (EPhyL), Instituto Antofagasta (IA), Universidad de Antofagasta, Antofagasta 1271155, Chile; fredi.cifuentes@uantof.cl
[7] Departamento Biomédico, Facultad Ciencias de la Salud, Universidad de Antofagasta, Antofagasta 1271155, Chile; fredi.cifuentes@uantof.cl
* Correspondence: clpalaci@unap.cl (J.P.); adrian.paredes@uantof.cl (A.P.); Tel.:+56-57-2526910 (J.P.); +56-55-2513507 (A.P.)

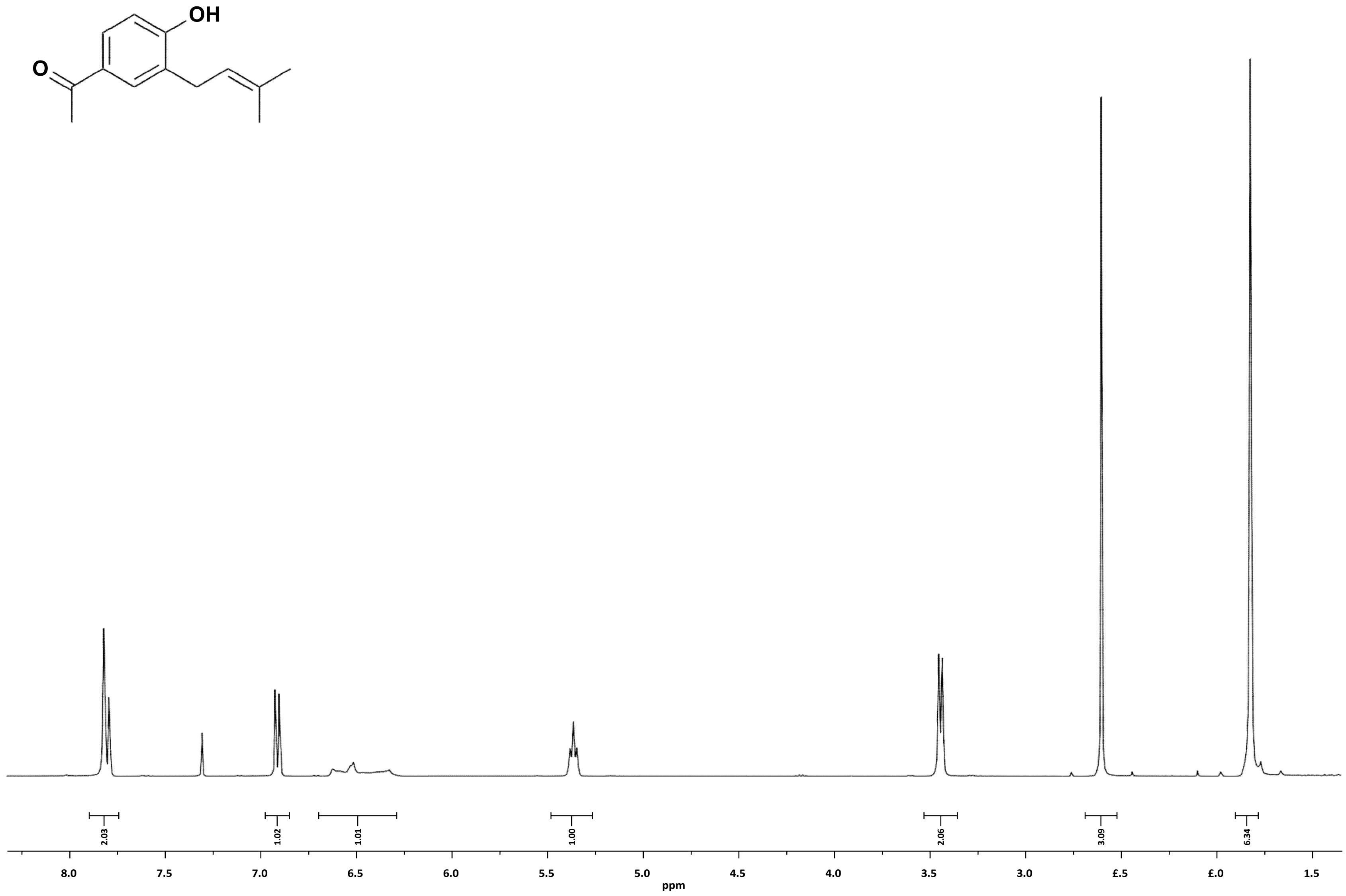

Figure S1. ^{1}H-NMR spectrum of metabolite-1

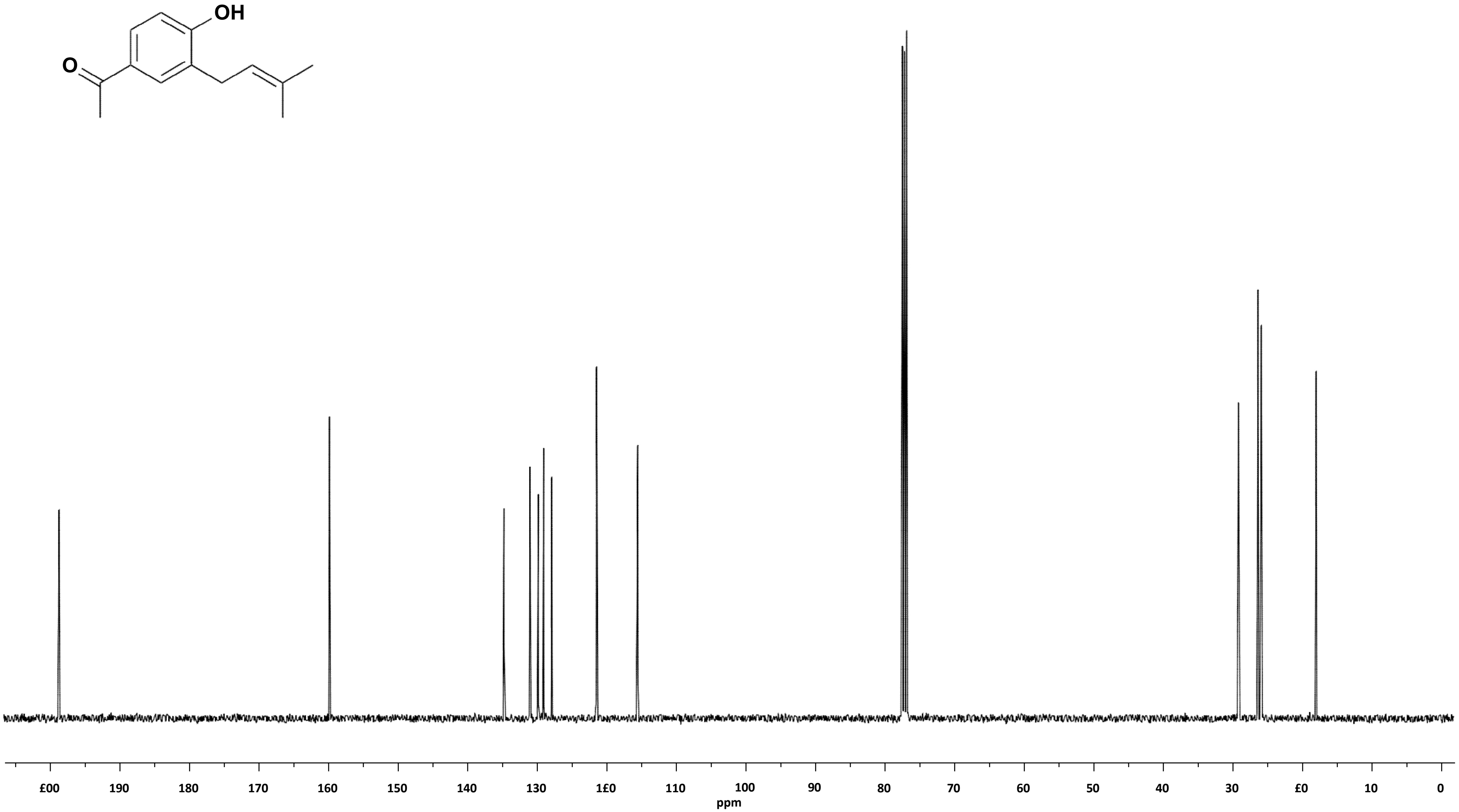

Figure S2. ^{13}C-NMR spectrum of metabolite-1

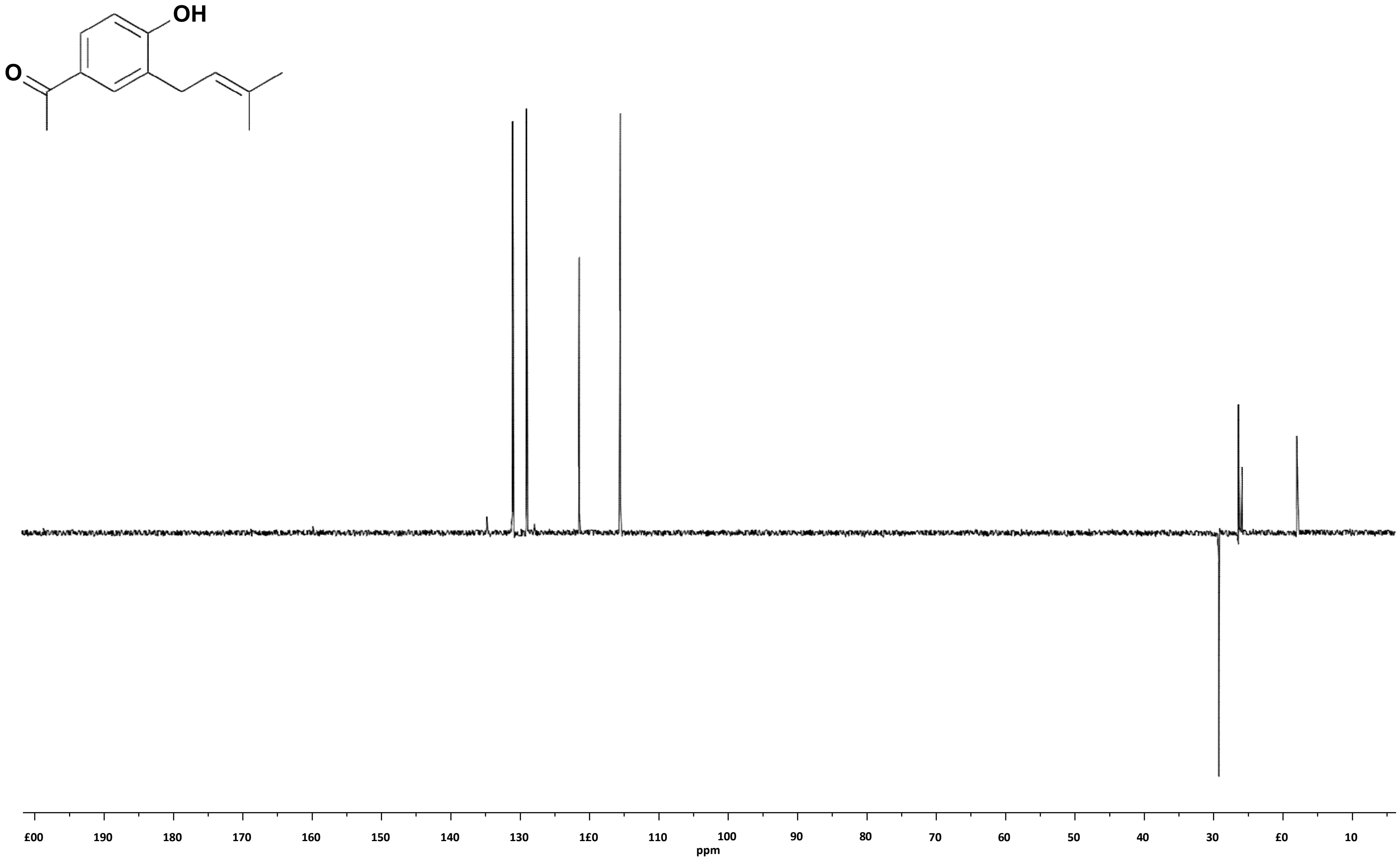

Figure S3. DEPT spectrum of metabolite-1

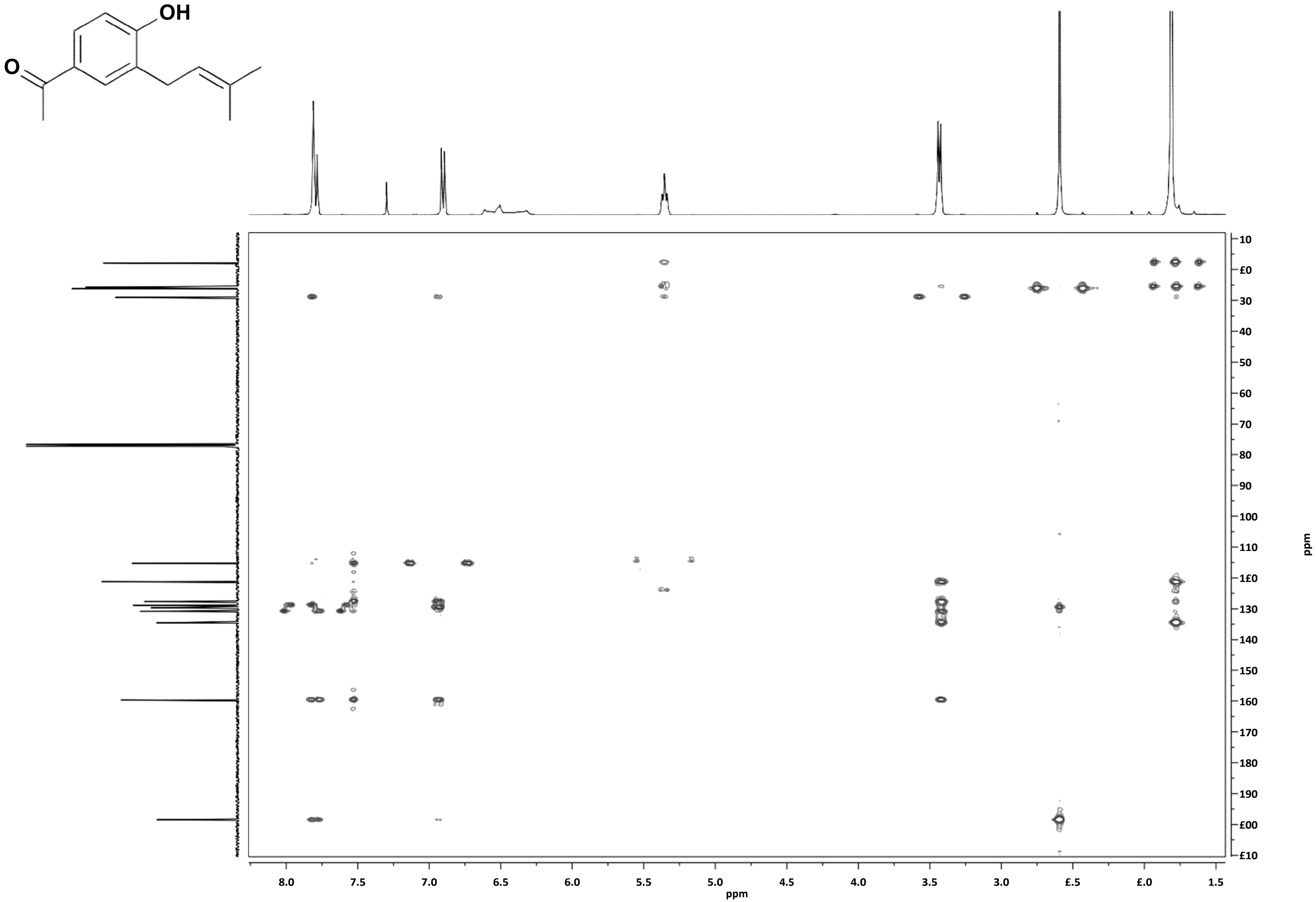

Figure S4. HMBC spectrum of metabolite-1

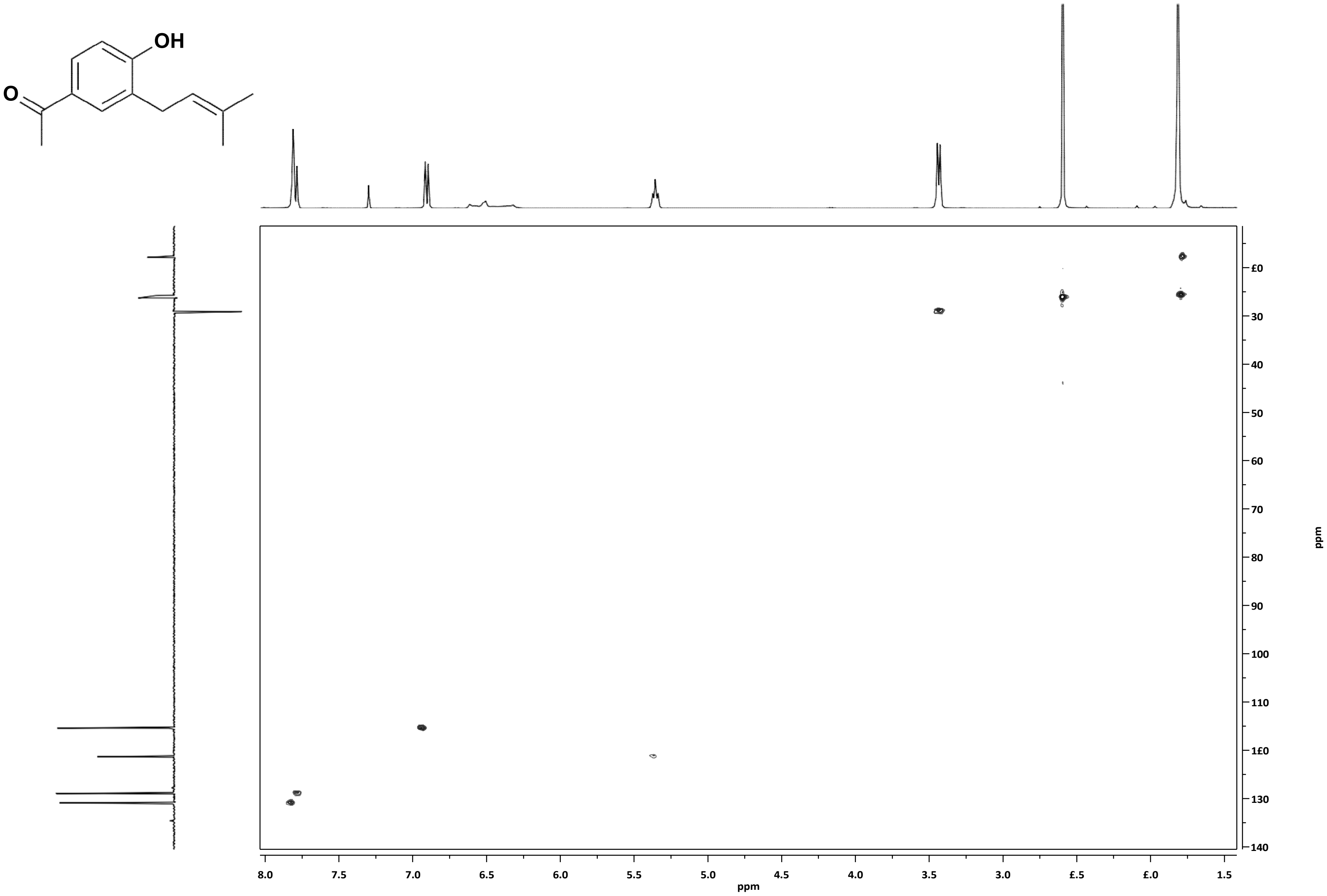

Figure S5. HMQC spectrum of metabolite-1

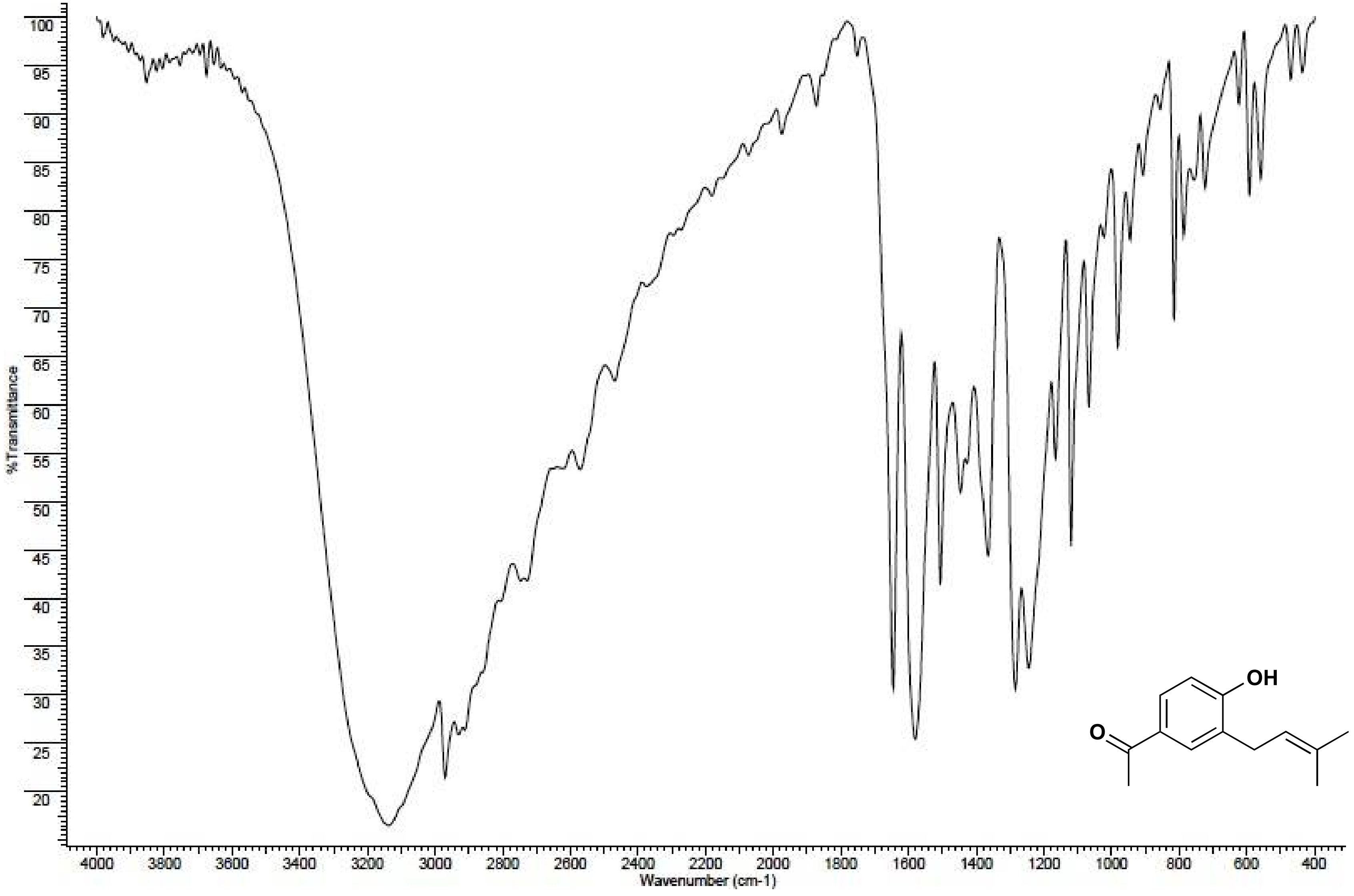

Figure S6. FT-IR spectrum of metabolite-1

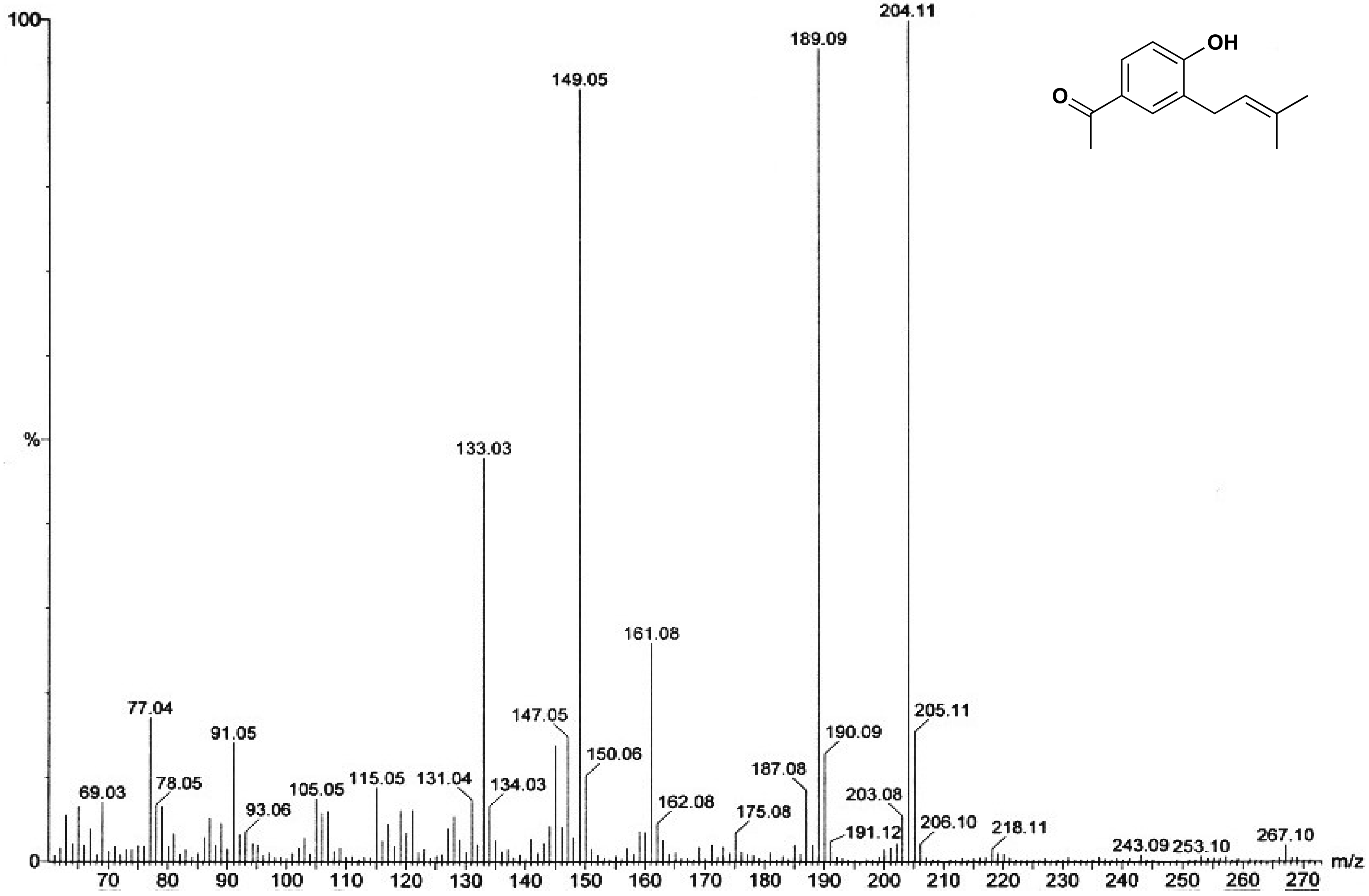

Figure S7. HR-MS spectrum of metabolite-1

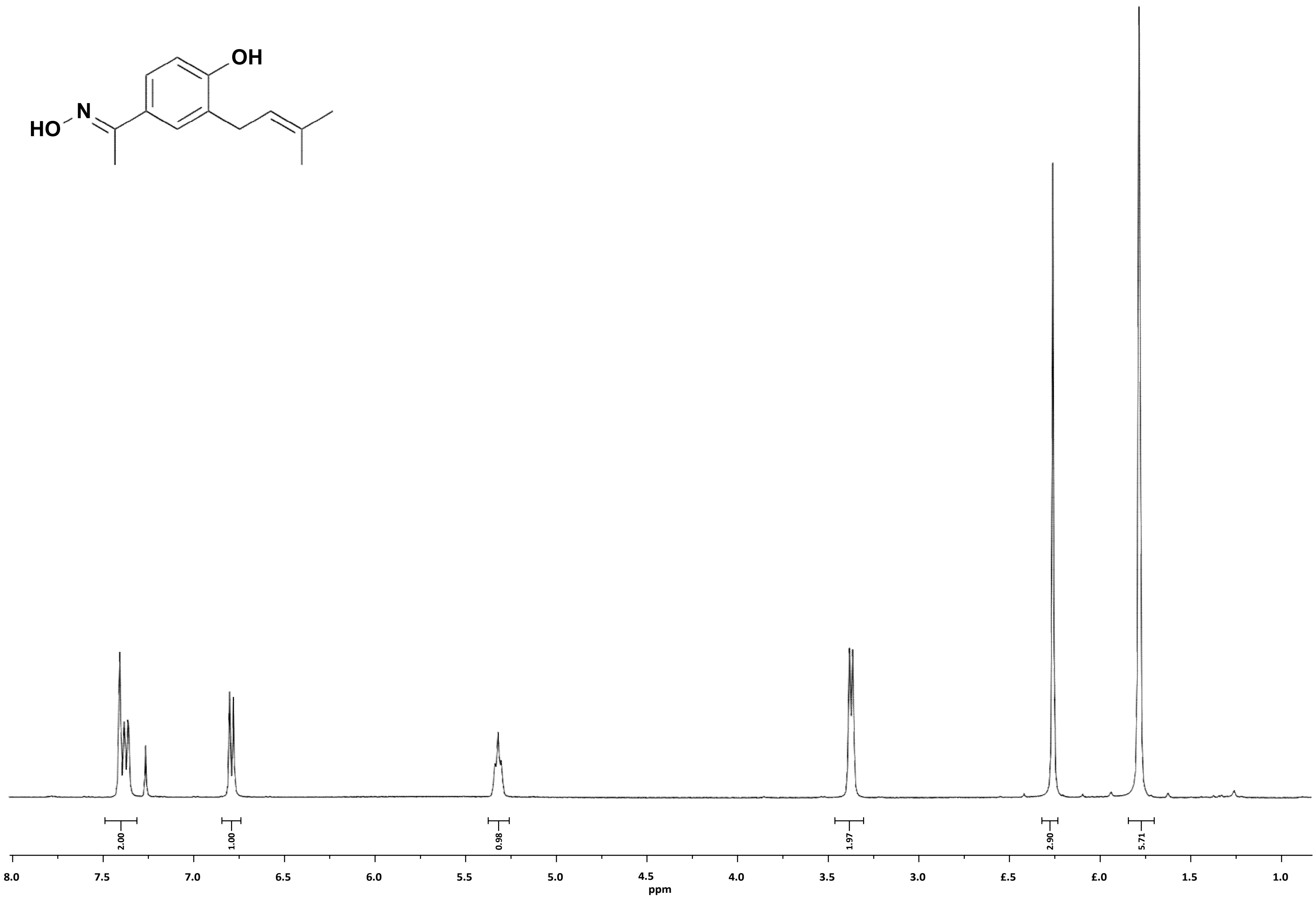

Figure S8. ^{1}H-NMR spectrum of oxime-1

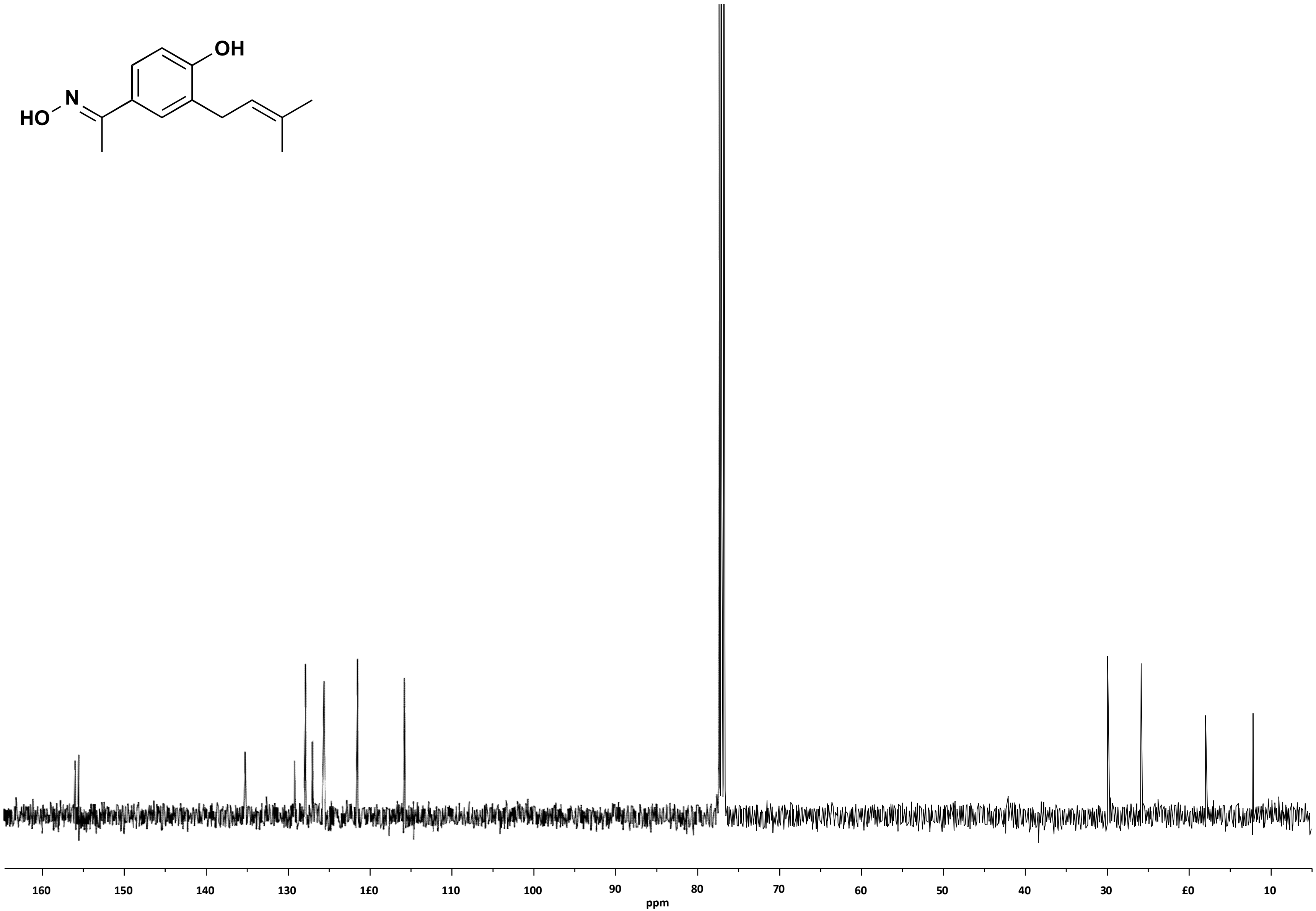

Figure S9. ^{13}C-NMR spectrum of oxime-1

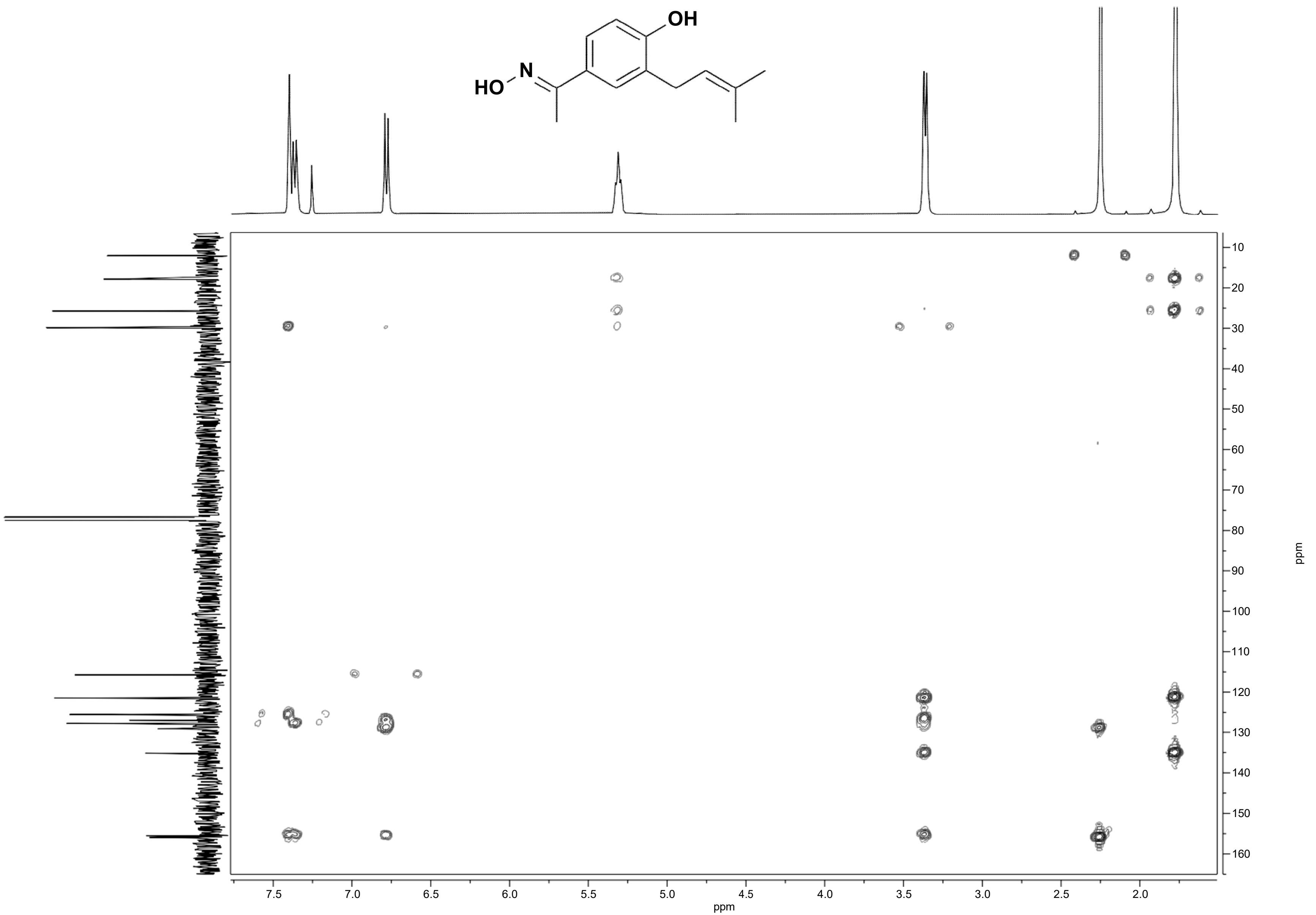

Figure S10. HMBC spectrum of oxime-1

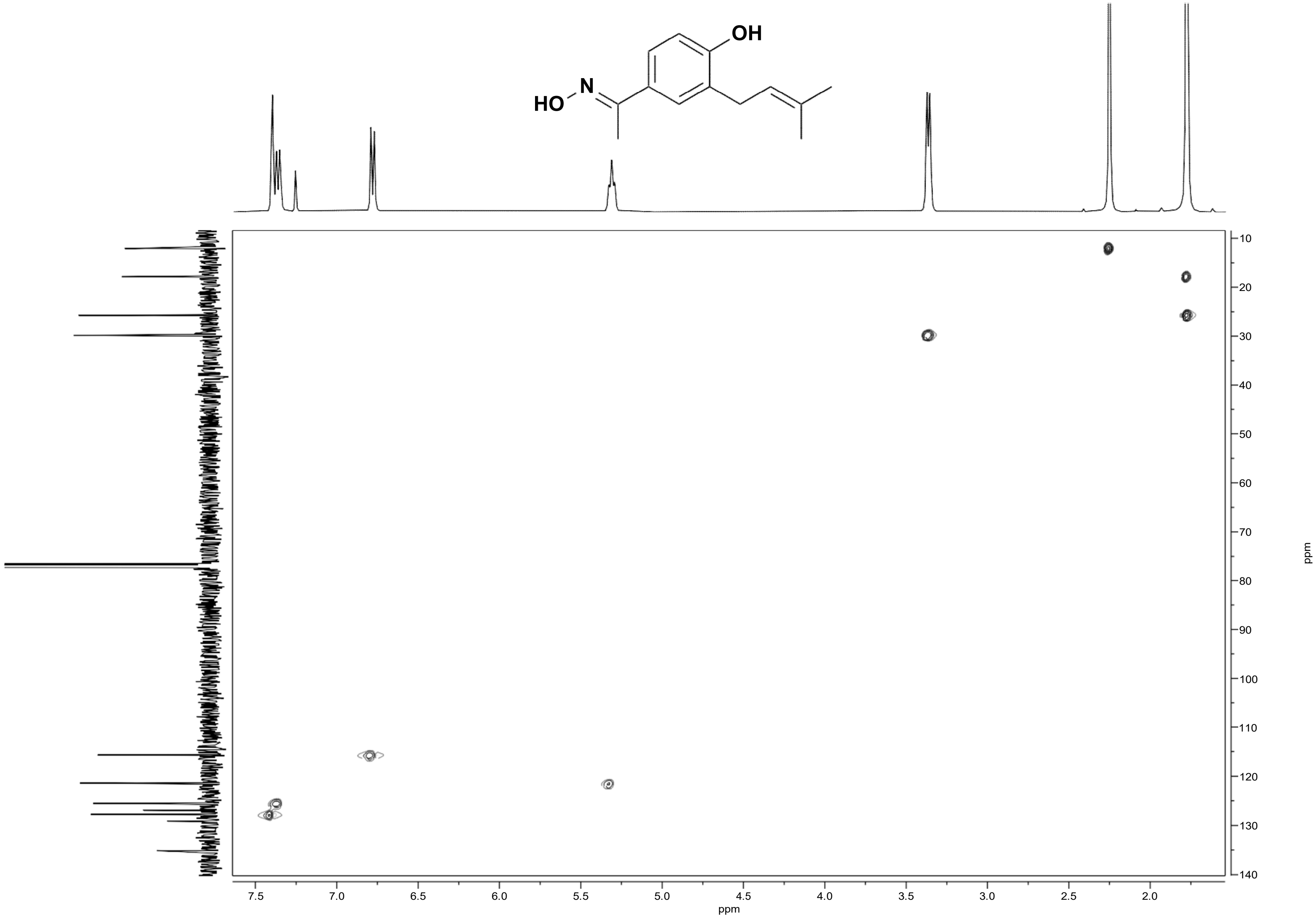

Figure S11. HSQC spectrum of oxime-1

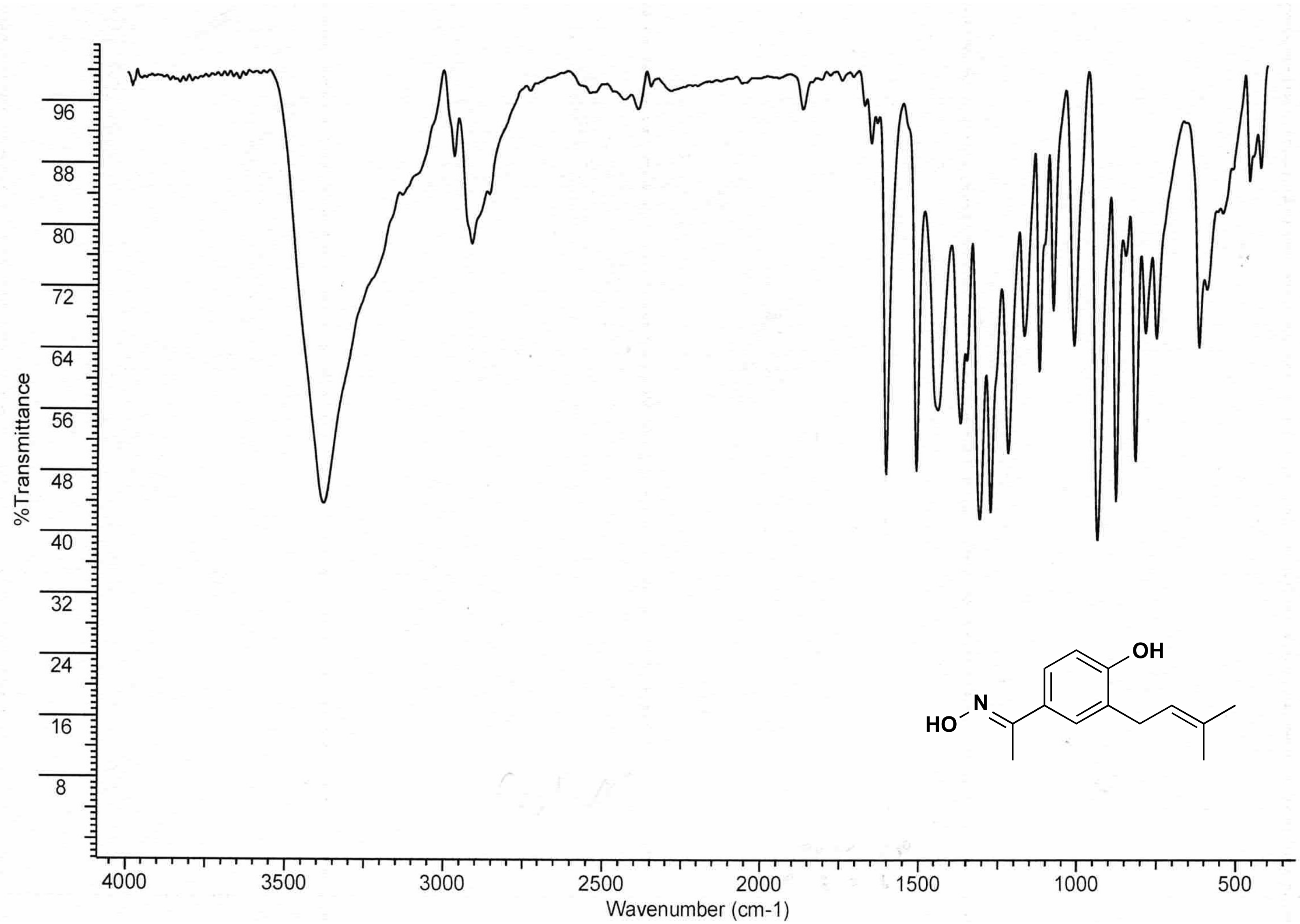

Figure S12. FT-IR spectrum of oxime-1

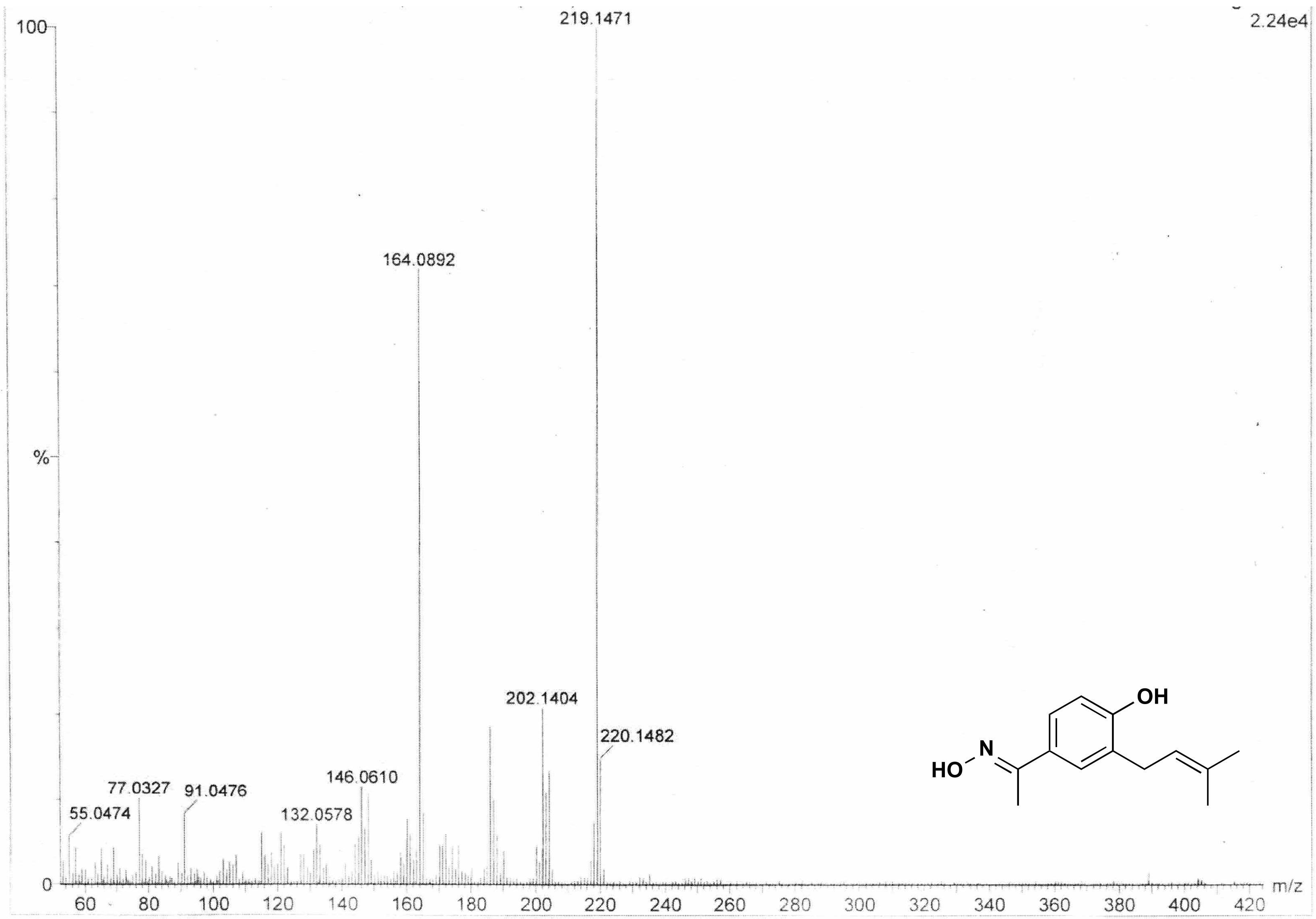

Figure S13 HR-MS spectrum of oxime-1
